# Mineral Composition and Antioxidant Potential in the Common Poppy (*Papaver rhoeas* L.) Petal Infusions

**DOI:** 10.1007/s12011-020-02134-7

**Published:** 2020-04-18

**Authors:** Janda Katarzyna, Jakubczyk Karolina, Kupnicka Patrycja, Bosiacki Mateusz, Gutowska Izabela

**Affiliations:** 1 Department of Human Nutrition and Metabolomics, Pomeranian Medical University in Szczecin, 24 Broniewskiego Street, 71-460 Szczecin, Poland; 2 Department of Biochemistry, Pomeranian Medical University in Szczecin, 71 Powstańców Wlkp. Street, 70-111 Szczecin, Poland; 3 Department of Functional Diagnostics and Physical Medicine, Pomeranian Medical University in Szczecin, 54 Żołnierska Street, 71-210 Szczecin, Poland; 4 Department of Medical Chemistry, Pomeranian Medical University in Szczecin, 71 Powstańców Wlkp. Street, 70-111 Szczecin, Poland

**Keywords:** Common poppy, Infusion, Antioxidant potential, Polyphenol, Vitamin C, Mineral content

## Abstract

The flowers of the common poppy are used for medicinal purposes, both internally and externally. They are reported to have antispasmodic and antitussive properties, to alleviate inflammatory conditions and soothe anxiety-related digestive problems. The aim of the study was to determine the antioxidant potential and the content of vitamin C, polyphenols, and minerals in infusions made from the petals of the common poppy at different temperatures. The infusions were made at various temperatures (25 °C, 70 °C, 80 °C, and 90 °C). The antioxidant potential and the content of polyphenols and vitamin C were determined by spectrophotometric methods. The mineral content was determined using the ICP-OES method. The total polyphenol content ranged from 135.2 to 137.24 ppm and that of vitamin C—from 15.47 to 15.78 mg/100 mL. The temperature of the water used to make the infusions did not appear to have a significant effect on these parameters. The temperature did, however, significantly affect the antioxidant potential of the infusions—the highest antioxidant activity (71.21% DPPH inhibition) was observed in the infusion prepared using water at 80 °C. The infusions included in the study contained a number of minerals. No significant effect of temperature was found for the content of K, Zn, Cu, Fe, and Ni in the infusions. On the other hand, the content of Ca in the infusions was significantly correlated with the increasing temperature of the water. It was concluded that poppy petal infusions may serve as a valuable dietary supplement, providing antioxidants and minerals required by the human body to function properly.

**Electronic supplementary material:**

The online version of this article (10.1007/s12011-020-02134-7) contains supplementary material, which is available to authorized users.

## Introduction

The common poppy, also known as the red poppy (*Papaver rhoeas* L.), grows in Europe, North Africa, Western Asia, and Pakistan. It is an annual plant which thrives in sunny positions and alkaline soils and is resistant to environmental pollution [1, 2]. Young leaves and flowers are a popular traditional ingredient in foods and beverages, particularly in Turkey and other Mediterranean countries [2–4]. In modern food industry, fresh or dried flowers of the plant are used as ingredients in food preparations [5]. The plant has historically been used as a folk remedy in many countries across Europe and Asia [3, 6, 7]. Phytochemical studies showed that the leaves of *Papaver rhoeas* L. contain several flavonols, like quercetin, kaempferol, myricetin, and isorhamnetin, as well as minerals, like potassium, sodium, and calcium [1, 4]. The properties of poppy extracts include antispasmodic, antitussive, anti-inflammatory, and antimicrobial effects, also soothing anxiety-related digestive problems [2, 3, 6–9].

Minerals are indispensable in human nutrition, and their content in the body depends on their levels in the soil, air, drinking water, and nutrition. An excess or deficiency of any chemical element may induce adverse effects in the human body [10]. It is common knowledge that humans need large quantities of macroelements, such as magnesium and calcium, whereas trace elements, including K, Na, Cr, Fe, and Cu, are required in low concentrations for specific physiological functions [11]. Numerous studies have been carried out in recent years to investigate the mineral content and heavy metal contamination of teas, fruits, vegetables, herbs, and other foods [12–16]. Inductively coupled plasma optical emission spectrometers (ICP-OES) are often used to determine the concentrations of elements, e.g., in herbs and medicinal plants [17]*.* While many other techniques (e.g., AAS, ICP-MS) are used to determine the concentrations of specific elements, ICP-OES allows for fast, simultaneous measurement of many different elements (both macro and micro) in both aqueous and solid samples, providing adequate sensitivity.

There have been no studies on the antioxidant potential and mineral content of poppy petal infusions. Hence, the aim of this study was to determine the composition of antioxidants and minerals in common poppy petal infusions made with water at 25 °C, 70 °C, 80 °C, and 90 °C.

## Material and Methods

### Plant Material

Plant material in the form of flower petals of common poppy (*Papaver rhoeas* L.) was collected from locations in West Pomeranian Voivodeship in north-western Poland. Directly after picking, the petals were cleaned, then frozen at − 20 °C and lyophilized in Alpha 1-2 LD plus (pressure 0.735 mmHg, temperature − 20 °C). The dried petals were homogenized (using FOSS Food Homogenizer 2094) and used to prepare infusions.

### Infusion Preparation

To make an infusion, 0.5 g of petals was transferred to a conical flask, together with 100.0 mL of water at a given temperature (25 °C, 70 °C, 80 °C, and 90 °C). The temperatures of 70, 80, and 90 °C are most commonly used to prepare plant infusions. When boiling water is poured into a container, it cools down to 90 °C, and this is the highest temperature that can be used. Numerous scientific studies confirm that the selection of the right brewing temperature for the raw material may be important and may determine the health-promoting properties of the resulting infusion, which has also been demonstrated in this study [18–21]. The flask with the infusion was then closed and rotated at 180 rpm for 10 min. After brewing, the plant material was separated from the infusion by filtration. All infusions were prepared in triplicate.

### Determination of Vitamin C Content

The determination of vitamin C content was carried out according to Wolska et al. [18]. In this method, 2,6-dichlorophenoloindophenol (2,6-DCPIP) is added to the sample; it reacts with vitamin C, and after extraction with xylene; its excess is determined spectrophotometrically. Vitamin C concentration was expressed in milligrams of vitamin C per 100.0 mL of infusion (mg/100.0 mL).

### Determination of Antioxidant Activity

The antioxidant activity of samples was measured with the spectrophotometric method using synthetic radical DPPH (2,2-diphenyl-1-picrylhydrazyl, Sigma). The antioxidant potential (antioxidant activity, inhibition) of the tested solutions was expressed as the percentage of DPPH inhibition [19].

### Determination of Polyphenol Content

Polyphenol content was assessed using the Folin-Ciocalteu reagent. A total of 5.0 mL of a 10% Folin-Ciocalteu solution and 1.0 mL of the test sample were successively introduced into a vial. The sample was shaken vigorously, and after 5 min, 4.0 mL of 7.5% Na_2_CO_3_ solution was added. The prepared solution was incubated for 60 min at room temperature. The reference solution was prepared the same way, but with distilled water added instead of the tested sample. Absorbance at 765 nm was measured. Polyphenol content (ppm) was calculated from the calibration curve plotted using gallic acid as the reference standard.

The content of polyphenols and vitamin C and antioxidant activity were determined using an Agilent 8453 UV-Vis spectrophotometer. All assays were performed in triplicate.

### Determination of the Mineral Content

#### Sample Preparation

The samples were mineralized using the CEM MARS 5 microwave digestion system. The sample volume was 0.8 mL. The samples were transferred to clean polypropylene tubes. A total of 0.6 mL of 65% HNO_3_ (Suprapur, Merck) was added to each vial, and each sample was allowed 30-min pre-reaction time in the clean hood. At the end of the pre-reaction time, 0.6 mL of non-stabilized 30% H_2_O_2_ solution (Suprapur, Merck) was added to each vial. After all reagents were added, the samples were placed in special Teflon vessels and heated in the microwave digestion system for 35 min at 180 °C (15-min ramp to 180 °C and maintained at 180 °C for 20 min). At the end of digestion, all samples were removed from the digestion oven and allowed to cool to room temperature. In the clean hood, samples were transferred to acid-washed 15-mL polypropylene sample tubes. A further tenfold dilution was performed prior to ICP-OES measurement. The volume of 1 mL was taken from each digest. The samples were spiked with an internal standard to provide a final concentration of 0.5 mg/L yttrium, 1 mL of 1% Triton (Triton X-100, Sigma), and diluted to a final volume of 10 mL with 0.075% nitric acid (Suprapur, Merck). Blank samples were prepared by adding concentrated nitric acid (500 μL) to tubes without the sample and subsequently diluted in the same manner as described above. Multi-element calibration standards (ICP multi-element standard solution IV, Merck) were prepared with different concentrations of inorganic elements in the same manner as in blanks and samples. Deionized water (Direct Q UV, Millipore, approximately 18.0 MΩ) was used in the preparation of all solutions.

#### Sample Determination

All samples were transferred into tubes and stored at − 20 °C until processed. Samples were analyzed using inductively coupled plasma optical emission spectrometry (ICP-OES, ICAP 7400 Duo, Thermo Scientific), which is often utilized to measure the concentrations of mineral nutrients as well as heavy metals and allows for simultaneous measurements of many different elements, also in plant samples [17, 22]. ICP-OES with a concentric nebulizer and cyclonic spray chamber was used to determine the content of micro- and macroelements. The analysis was performed in both radial and axial modes.

Validation was performed by evaluating the following: NIST SRM 8414 reference material (National Institute of Standards and Technology, USA), limit of detection (LOD), and the recovery of internal standard (yttrium). To eliminate possible interference, the emission lines were selected empirically in pilot measurement. This model of validation is often used in ICP-OES studies, also those regarding plant samples [22]. The recovery of Y was within 89–105%. The *R*^2^ values for all standard curves were in the range between 0.998 and 0.100.

### Statistical Analysis

In all the experiments, three samples were analyzed, and all the assays were carried out at least in triplicate. The statistical analysis was performed using the Stat Soft Statistica 13.0 and Microsoft Excel 2010. The results are expressed as mean values ± standard deviation (SD).

To assess the differences between examined parameters, one-way analysis of variance (ANOVA) with Tukey’s post hoc test was used. Differences were considered significant at *p* ≤ 0.05. To control type I errors, the false discovery rate (FDR) approach was used. The calculations were performed using the p.adjust function of the stats package in R (https://cran.r-project.org).

## Results

The total polyphenol content in the infusions ranged from 135.2 to 137.24 ppm (Table 1), and the differences depending on the temperature of water used to prepare the infusion were statistically insignificant (FDR *p* ≥ 0.7777).

**Table 1:** Antioxidant potential (DPPH), content of polyphenols, vitamin C, and elements in common poppy petal infusions in dependence on temperature (*designates significance at *p* ≤ 0.05)

Parameter (mean ± SD)	Temperature (°C)			
	25C° - ^a^	70C° - ^b^	80C° - ^c^	90C° - ^d^
DPPH (% inhibition)	61.48 (±1.34)*^b,c,d^	65.91 ± (0.23)*^a,c,d^	71.21 (± 4.93)*^a,b^	70.25 (± 1.56)*^a,b^
Polyphenols (ppm)	136.07 (± 0.005)	135.20 (± 5.14)	137.24 (± 4.78)	135.87 (± 2.42)
Vitamin C (mg/100 g)	15.78 (± 0.60)	15.50 (± 0.15)	15.72 (± 0.22)	15.47 (± 0.04)
Ca (ppm)	1.60 (± 0.08)*^b,c,d^	1.96 (± 0.22)*^a,d^	2.07 (± 0.13)*^a,d^	2.49 (± 0.31)*^a,b,c^
Mg(ppm)	6.55 (± 0.29)*^b,c^	7.63 (± 0.69)*^a^	7.78 (± 0.70)*^a^	7.36 (± 0.23)
P (ppm)	17.377 (± 0.939)	19.844 (± 2.056)	20.099 (± 1.967)	17.724 (± 0.527)
K (ppm)	261.785 (± 15.692)	287.806 (± 27.506)	282.081 (± 24.770)	257.378 (± 7.792)
Na (ppm)	5.239 (± 2.727)*^b,c^	2.890 (± 0.182)*^a^	2.550 (± 0.486)*^a^	3.389 (± 0.415)
Mn (ppm)	0.037 (± 0.002)*^b,c,d^	0.044 (± 0.004)*^a^	0.046 (± 0.004)*^a^	0.047 (± 0.0004)*^a^
Zn (ppm)	0.192 (± 0.009)	0.241 (± 0.018)	0.202 (± 0.021)	0.216 (± 0.070)
Cu (ppm)	0.050 (± 0.009)	0.057 (± 0.0009)	0.048 (± 0.009)	0.065 (± 0.050)
Fe (ppm)	0.288 (± 0.045)	0.282 (± 0.006)	0.286 (± 0.018)	0.293 (± 0.037)
Mo (ppm)	0.011 (± 0.006)*^d^	0.010 (± 0.003)*^d^	0.012 (± 0.005)*^d^	0.021 (± 0.001*)*^a,b,c^
Cr (ppm)	0.026 (± 0.001)*^d^	0.027 (± 0.002)	0.029 (± 0.0008)	0.030 (± 0.003)*^a^
Si (ppm)	1.135 (± 0.078)*^b,c,d^	1.513 (± 0.134)*^a,d^	1641 (± 0.1423)*^a^	1842 (± 0.208)*^a,b^
Ni (ppm)	0.021 (± 0.003)	0.020 (± 0.002)	0.021 (± 0.005)	0.030 (± 0.014)
Sr (ppm)	0.016 (± 0.001)	0.015 (± 0.002)	0.013 (± 0.0003)	0.015 (± 0.001)
Al (ppm)	0.400 (± 0.026)	0.420 (± 0.021)	0.396 (± 0.037)	0.483 (± 0.085)

The highest antioxidant potential (71.21% DPPH inhibition) was observed in the infusion prepared with water at 80 °C. The lowest value (61.48% DPPH inhibition) was determined in the infusion made at 25 °C (Table 1). In the majority of cases, the differences in the antioxidant potential of the respective infusions were statistically significant (FDR *p* < 0.05), except for those made at 80 and 90 °C—the difference between these values was statistically insignificant (FDR *p* = 0.9229).

The content of vitamin C in the poppy infusions was similar irrespective of the temperature at which the infusion was made, ranging from 15.47 to 15.78 mg/100 ml, for the temperatures of 90 °C and 25 °C, respectively (Table 1). The temperature of the water used to make the infusions did not have a significant effect on the content of vitamin C (FDR *p* > 0.3922).

The level of Ca in the infusions increased with the rising temperatures of water used to make them (*R*^2^ = 0.9565). The lowest calcium level was observed in the infusion obtained at 25 °C (1.6 mg/L), and the highest—at 90 °C (2.5 mg/L) (Table 1). It was demonstrated that temperature has a significant effect on the Ca content in the infusions (FDR *p* ≤ 0.05). No significant differences were observed as to the content of the Ca between the infusion made with water at 70 vs. 80 °C (FDR *p* = 0.8062).

The content of Mg ranged from 6.5 to 7.8 mg/L, respectively, for infusions made at 25 °C and 80 °C (Table 1). Statistically significant differences were observed between the values obtained for infusions made at 25 vs. 70 °C (FDR *p* = 0.014835) and 25 vs. 80 °C (FDR *p* = 0.00987).

The content of P ranged from 17.37 to 20.09 mg/L, respectively, for infusions made at 25 °C and 80 °C (Table 1). No statistically significant differences were found for the infusions made at 25 vs. 70 °C (FDR *p* = 0.07383) and 25 vs. 80 °C (FDR *p* = 0.07383).

The content of K was in the range of 257.38–287.81 mg/L (Table 1), depending on the preparation temperature, but the differences between the respective values were statistically insignificant (*p* ≥ 0.0785).

The content of Na in the poppy infusions ranged from 2.55 to 5.23 mg/L, respectively, for infusions made at 70 °C and 25 °C (Table 1), but the differences were not statistically significant (FDR *p* > 0.05).

The content of Mn in the infusions went up with the rising preparation temperatures (*R*^2^ = 0.8126) and amounted to between 0.037 and 0.047 mg/L, corresponding to infusions made at 25 °C and 90 °C, respectively (Table 1). Statistically significant differences were observed for the lowest value and those obtained at the other temperatures: 25 vs. 70 °C (FDR *p* = 0.0027), 25 vs. 80 °C (FDR *p* = 0.000465), and 25 vs. 90 °C (FDR *p* = 0.000465).

The content of Zn ranged from 0.19 to 0.24 mg/L, respectively, for infusions made at 25 °C and 70 °C (Table 1). Temperature was not observed to have a significant effect on the content of that mineral in the infusions (*p* ≥ 0.1459).

The content of Cu ranged from 0.047 to 0.065 mg/L, respectively, for infusions made at 70 °C and 90 °C (Table 1). Temperature was not observed to have a significant effect on the content of that mineral in the infusions (*p* ≥ 0.6528).

The content of Fe was similar across the board, ranging from 0.282 to 0.293 mg/L, respectively, for infusions made at 70 °C and 90 °C (Table 1). Temperature was not observed to have a significant effect on the content of that mineral in the infusions (*p* ≥ 0.9292).

The content of Mo in the studied infusions ranged from 0.011 to 0.021 mg/L, respectively, for infusions made at 70 °C and 90 °C (Table 1). Statistically significant differences were found with respect to the Mo content in the infusion prepared at 90 °C and the others: 90 vs. 25 °C (FDR *p* = 0.00771), 90 vs. 70 °C (FDR *p* = 0.0048), and 90 vs. 80 °C (FDR *p* = 0.01309).

The content of Cr increased together with the temperatures at which the infusions were prepared (*R*^2^ = 0.9179) and amounted to between 0.264 and 0.302 mg/L, corresponding to the infusions made at 25 °C and 90 °C, respectively (Table 1), but the differences were not statistically significant (FDR *p* > 0.05).

It was observed that for infusions prepared at higher temperatures, the content of Si also increased (*R*^2^ = 0.9514). Its levels ranged from 1.135 to 1.842 mg/L, respectively, for infusions made at 25 °C and 90 °C (Table 1). The differences in the content of that mineral were not statistically significant only with regard to the infusions made at 80 vs. 70 °C (*p* = 0.4580) and 80 vs. 90 °C (*p* = 0.1171).

The content of Ni ranged from 0.019 to 0.0301 mg/L, respectively, for infusions made at 70 °C and 90 °C (Table 1). Statistically significant effects of infusion water temperatures on the content of that mineral were not observed (*p* ≥ 0.1313).

The content of Sr ranged from 0.013 to 0.016 mg/L, respectively, for infusions made at 80 °C and 25 °C (Table 1), but the differences were not statistically significant (FDR *p* > 0.05).

The content of Al amounted to between 0.396 and 0.483 mg/L, for the infusions made at 80 °C and 90 °C, respectively (Table 1), but no statistically significant differences were observed (FDR *p* > 0.05).

The concentrations of other heavy metals, such as Pb, Cd, and As, were also measured in this study; however, the content of those elements in digested samples failed to reach the detection limit (LOD) of ICP-OES (Pb LOD 0.003 mg/L, Cd LOD 0.0003 mg/L, As LOD 0.0013 mg/L).

### Correlations

The data were checked for relationships between the temperature and studied parameters. A significant positive correlation was found between the temperature and antioxidant potential of infusions (*r* = 0.789), meaning that the higher the water temperature used to make infusions, the higher the antioxidant potential of those infusions.

Significant positive correlations were also observed between temperature and the levels of the following minerals: Ca (*r* = 0.793), Mg (*r* = 0.599), Mn (*r* = 0.817), Mo (*r* = 0.410), Cr (*r* = 0.507), and Si (*r* = 0.869). Only in the case of Na was a significant negative correlation found (*r* = − 0.547), meaning that the higher the water temperature used to prepare the infusion, the less Na it contained.

Tables 2, 3, 4, 5, 6, 7, 8 and 9 present the statistically significant correlations between the content of polyphenols and vitamin C vs. the mineral content of the infusions, as well as the relationships between the minerals themselves in infusions made at different temperatures. It was demonstrated that the correlations between the parameters included in the study are highly varied, depending on the infusion water temperature.

**Table 2:** Statistically significant (at *p* ≤ 0.05) correlation (*r*) between mineral content and polyphenols and vitamin C in common poppy petal infusion prepared in temperature 25 °C

Correlations (*r*) between analyzed parameters		
	Positive	Negative
Polyphenols and	Fe (*r* = 0.82)	Ca (*r* = − 0.85); Cu (*r* = − 0.93); Si (*r* = − 0.99)
Vitamin C and	Mg (*r* = 0.96); Mn (*r* = 0.99); P (*r* = 0.94); K (*r* = 0.97); Fe (*r* = 0.87); Ni (*r* = 0.95); Sr (*r* = 0.97)	Ca (*r* = − 0.84); Mo (*r* = − 0.97)

**Table 3:** Statistically significant (at *p* ≤ 0.05) correlation (*r*) between elements in common poppy petal infusion prepared in temperature 25 °C

Correlations (*r*) between elements		
	Positive	Negative
Ca and	Si (*r* = 0.90)	Mn (*r* = −0.89); Fe (*r* = − 0.99); Ni (*r* = − 0.97); Sr (*r* = − 0.94)
Mg and	Mn (*r* = 0.93); P (*r* = 0.99); K (*r* = 1.000); Zn (*r* = 0.87)	Mo (*r* = − 0.999)
P and	K (*r* = 0.99); Zn (*r* = 0.90); Na (*r* = 0.84); Sr (*r* = 0.84)	Mo (*r* = − 0.99)
K and	Zn (*r* = 0.86); Ni (*r* = 0.83); Sr (*r* = 0.83)	Mo (*r* = − 1.000)
Na and	Cr (*r* = 0.97)	Al (*r* = − 0.99)
Mn and	P (*r* = 0.90); K (*r* = 0.94); Fe (*r* = 0.92); Ni (*r* = 0.97); Sr (*r* = 0.99)	Mo (*r* = − 0.94)
Zn and	Na (*r* = 0.99); Cr (*r* = 0.92)	Mo (*r* = − 0.856); Al (*r* = − 0.97)
Cu and	Cr (*r* = 0.899); Si (*r* = 0.883)	Al (*r* = − 0.821)
Fe and	Ni (*r* = 0.98); Sr (*r* = 0.96)	Si (*r* = − 0.87)
Mo and		Ni (*r* = − 0.84); S*r* (*r* = − 0.89)
Cr and		Al (*r* = − 0.98)
Ni and	Sr (*r* = 0.995)

**Table 4:** Statistically significant (at *p* ≤ 0.05) correlation (*r*) between mineral content and polyphenols and vitamin C in common poppy petal infusion prepared in 70 °C

Correlations (*r*) between analyzed parameters		
	Positive	Negative
Polyphenols and		Ca (*r* = − 0.99); Fe (*r* = − 0.90); Cr (*r* = − 0.99); Ni (*r* = − 0.99); Si (*r* = − 0.96); Al (*r* = − 0.98)
Vitamin C and	Zn (*r* = 0.85); Cu (*r* = 0.99); Na (*r* = 0.97)

**Table 5:** Statistically significant (at *p* ≤ 0.05) correlation (*r*) between elements in common poppy petal infusion prepared in 70 °C

Correlations (*r*) between elements		
	Positive	Negative
Ca and	Fe (*r* = 0.92); Cr (*r* = 0.99); Ni (*r* = 1.000); Si (*r* = 0.97); Al (*r* = 0.97)	
Mg and	Mn (*r* = 0.99); P (*r* = 0.99); K (*r* = 0.99); Zn (*r* = 0.99)	Sr (*r* = − 0.86)
P and	K (*r* = 1.000); Zn (*r* = 0.95)	Sr (*r* = − 0.93)
K and	Zn (*r* = 0.96)	Sr (*r* = − 0.93)
Mn and	P (*r* = 0.99); K (*r* = 0.99); Zn (*r* = 0.98)	Sr (*r* = − 0.89)
Zn and	Cu (*r* = 0.84)	
Cu and	Na (*r* = 0.98)	
Fe and	Na (*r* = 0.82); Cr (*r* = 0.89); Ni (*r* = 0.93); Si (*r* = 0.99)	
Cr and	Ni (*r* = 0.99); Si (*r* = 0.95); Sr (*r* = 0.83); Al (*r* = 0.98)	
Ni and	Si (*r* = 0.98); Al (*r* = 0.95)	
Sr and	Al (*r* = 0.91)	
Al and	Si (*r* = 0.880)

**Table 6:** Statistically significant (at *p* ≤ 0.05) correlation (*r*) between mineral content and polyphenols and vitamin C in common poppy petal infusion prepared in temperature 80 °C

	Positive	Negative
Polyphenols and		Fe (*r* = −0.97); Sr (*r* = − 0.88); Al (*r* = − 0.99)
Vitamin C and	Cr (*r* = 0.99); Sr (*r* = 0.89)	Ca (*r* = − 0.99); Zn (*r* = − 0.98); Cu (*r* = − 0.97); Na (*r* = − 0.96); Ni (*r* = − 0.93)

**Table 7:** Statistically significant (at *p* ≤ 0.05) correlation (*r*) between elements in common poppy petal infusion prepared in temperature 80 °C

	Positive	Negative
Ca and	Zn (*r* = 0.99); Cu (*r* = 0.99); Na (*r* = 0.93); Ni (*r* = 0.89)	Cr (*r* = − 0.98); Ni (*r* = 0.89); Sr (*r* = − 0.94)
Mg and	Mn (*r* = 0.99); P (*r* = 0.99); K (*r* = 0.99); Si (*r* = 0.87)	
Mn and	P (*r* = 0.99); K (*r* = 0.99); Si (*r* = 0.89)	
P and	K (*r* = 0.99); Ni (*r* = 0.81); Si (*r* = 0.86)	
K and	Ni (*r* = 0.81); Si (*r* = 0.86)	
Mn and	P (*r* = 0.90); K (*r* = 0.94); Fe (*r* = 0.92); Ni (*r* = 0.97); Sr (*r* = 0.99)	Mo (*r* = − 0.94)
Zn and	Cu (*r* = 0.99); Na (*r* = 0.91); Ni (*r* = 0.87)	Cr (*r* = − 0.97); Sr (*r* = − 0.96)
Cu and	Na (*r* = 0.89); Ni (*r* = 0.84)	Cr (*r* = − 0.96); Sr (*r* = − 0.97)
Fe and	Al (*r* = 0.99); Si (*r* = 0.90)	
Na and	Ni (*r* = 0.99)	Cr (*r* = − 0.98)
Cr and	Sr (*r* = 0.86)	Ni (*r* = − 0.96)
Al and	Si (*r* = 0.86)

**Table 8:** Statistically significant (at *p* ≤ 0.05) correlation (*r*) between mineral content and polyphenols and vitamin C in common poppy petal infusion prepared in temperature 90 °C

Correlations (*r*) between analyzed parameters		
	Positive	Negative
Polyphenols and	Zn (*r* = 0.98); Cu (*r* = 0.85); Mo (*r* = 0.91); Ni (*r* = 0.86)	Si (*r* = − 0.891)
Vitamin C and		Mn (*r* = − 0.94); Sr (*r* = − 0.97)

**Table 9:** Statistically significant (at *p* ≤ 0.05) correlation (*r*) elements in common poppy petal infusion prepared in temperature 90 °C

Correlations (*r*) between elements		
	Positive	Negative
Ca and	Mg (*r* = 0.96); P (*r* = 0.99); K (*r* = 0.99); Na (*r* = 0.99); Si (*r* = 0.82)	Cu (*r* = − 0.86); Fe (*r* = − 0.99); Cr (*r* = − 0.91); Ni (*r* = − 0.85); Al (*r* = − 0.99)
Mg and	P (*r* = 0.94); K (*r* = 0.94); Na (*r* = 0.97); Si (*r* = 0.94)	Zn (*r* = − 0.82); Cu (*r* = − 0.97); Fe (*r* = − 0.99); Mo (*r* = − 0.92); Ni (*r* = − 0.96); Al (*r* = − 0.98)
P and	K (*r* = 1.00); Na (*r* = 0.99)	Cu (*r* = − 0.82); Fe (*r* = − 0.98); Cr (*r* = − 0.94); Al (*r* = − 0.99)
K and	Na (*r* = 0.99)	Cu (*r* = − 0.83); Fe (*r* = − 0.98); Cr (*r* = − 0.93); Ni (*r* = − 0.81); Al (*r* = − 0.99)
Mn and	Sr (*r* = 0.88)	Cr (*r* = − 0.94)
Zn and	Cu (*r* = 0.93); Mo (*r* = 0.97); Ni (*r* = 0.94)	Si (*r* = − 0.96)
Cu and	Fe (*r* = 0.93); Mo (*r* = 0.99); Ni (*r* = 1.00); Al (*r* = 0.90)	Na (*r* = − 0.88); Si (*r* = − 0.99)
Fe and	Mo (*r* = 0.86); Cr (*r* = 0.84); Ni (*r* = 0.92); Al (*r* = 0.99)	Na (*r* = − 0.99); Si (*r* = − 0.89)
Na and	Si (*r* = 0.84)	Cr (*r* = − 0.89); Ni (*r* = − 0.87); Al (*r* = − 0.99)
Mo and	Ni (*r* = 0.99); Al (*r* = 0.83)	Si (*r* = − 0.99)
Ni and	Al (*r* = 0.89)	
Cr and	Al (*r* = 0.87)	

In the infusions made at 25 °C, significant correlations were found between polyphenol and mineral content: a positive correlation between polyphenol content and Fe, and negative correlations for polyphenols vs. Ca, Cu, and Si (Table 2).

Strong positive correlations were also shown for vitamin C vs. Mg, Mn, P, K, Fe, Ni, and Sr and negative correlations with regard to the content of Ca and Mo (Table 2).

Statistically significant correlations between the levels of individual minerals in the infusions made at 25 °C are presented in Table 3.

In the infusions made at 70 °C, the correlations between polyphenol and mineral content were all inversely proportionate and observed for Ca, Fe, Cr, Ni, Si, and Al (Table 4). Conversely, only positive correlations were found for vitamin C with Zn, Cu, and Na (Table 4).

Statistically significant correlations between the levels of individual minerals in the infusions made at 70 °C are presented in Table 5.

In the infusions made at 80 °C, significant negative correlations were observed between polyphenol content and that of Fe, Sr, and Al (Table 6).

Positive correlations were demonstrated for vitamin C vs. the content of Cr and Sr and negative correlations between the content of that vitamin and those of Ca, Zn, Cu, Na, and Ni (Table 6).

Statistically significant correlations between the levels of individual minerals in the infusions made at 80 °C are presented in Table 7.

In the infusions made at 90 °C, significant positive correlations were found between polyphenol content and the amounts of Zn, Cu, Mo, and Ni, and negative correlations with regard to the Si content (Table 8). Correlations between the content of vitamin C and that of minerals were negative and found only for Mn and Sr (Table 8).

Statistically significant correlations between the levels of individual minerals in the infusions made at 90 °C are presented in Table 9.

## Discussion

### Antioxidant Potential

Papers documenting free radical scavenging ability of antioxidant substances, notably those found in specific flower species, are few and far between. On the other hand, there have been studies which demonstrated that flowers contain significantly higher quantities of bioactive substances with antioxidant properties compared with fruit. Pachlowska et al. [23], in a study comparing the flowers and fruit of several pumpkin varieties, demonstrated that flowers had more than 10 times the content of antioxidants found in fruit. The researchers showed the amount found in flowers ranged from 78.96 to 110.51 mg 100^−1^ FW (fresh weight), compared with as little as between 3.39 and 9.84 mg 100^−1^ FW in fruit. A similar association was demonstrated by Kołodziej and Drożdżal [24], who analyzed the flowers and fruit of elderberry. Elderflowers had a higher content of polyphenolic compounds (from 37.02 to 53.33 mg g^−1^ DM [dry mass]) compared with elderberries (from 26.84 to 44.8 mg g^−1^ DM) as well as greater antioxidant capacity than the fruit, which was confirmed using FRAP and DPPH assays. These studies prove that flowers may provide a more effective instrument than fruit in the battle against oxygen free radicals in the body.

The antioxidant potential of poppy flowers was investigated by Hasplova et al. [25]. In their study, a 5 mg/mL methanol extract made at 65 °C exhibited approx. 60% DPPH inhibition, which is comparable with the values obtained in this study of 60–70% for the 0.5% water infusion. Higher parameters were obtained by Kostic et al. [26], where the aqueous extract of poppy petals produced 90% inhibition. The difference may have been due to the methodology or, even more so, the concentrations of the studied extracts and the source of the material.

Studies of different infusions (1%) demonstrated that the highest antioxidant potential out of all the products included in the study was observed in the dried form of rosehip (80.1%), chokeberry (63.4%), hibiscus (57.9%), and raspberry (40.7%) [27]. Studies also showed that commercially available herbal infusions exhibited varied levels of antioxidant activity, from 61.8 to 67.1% DPPH inhibition, depending on their composition and manufacturer [27]. Our own study demonstrated that poppy flower infusions had the highest antioxidant activity at the temperature of 80 °C—71.21%, which is just 7.33% less than the dried rosehip infusion, and on the other hand 14.56% more than the infusions of dried chokeberry [28], confirming the value of the edible flowers investigated in this study as a source of antioxidants. Please note as well that the concentration of the poppy infusions used in this study was only 0.5%, so the material itself has a very high potential for scavenging free radicals.

It is also worth stressing that the antioxidant potential of poppy infusions is only slightly lower than that of coffee or green tea, which are regarded as powerful antioxidants [29, 30].

The high antioxidant potential of poppy infusions is due to the presence of compounds capable of scavenging free radicals. Such compounds found in poppy include lutein, β-carotene [31], and the already-mentioned polyphenols. Many scholars observed a strong positive correlation between the antioxidant potential and overall polyphenolic content in plant material, which indicates that these compounds are closely linked to this parameter.

Fresh poppy leaves contain 25.86 mg GAE/g extract of total phenolics and 1.87 mg/100 g FW of total tocopherols [32]. Scholars also measured the total polyphenol content in the poppy flower extract, obtaining 19.9 mg of gallic acid from a gram of fresh flowers. The value for this parameter observed in this study (135–137 ppm), once converted into the same units, is lower, but one should remember that it refers to the polyphenolic content in flower infusions, rather than in the flowers themselves [26]. The high polyphenolic content found in the studied infusions proves that the beverage is a rich source of biologically active compounds. Polyphenolics are credited with a broad range of health-promoting properties, including antioxidant, antimicrobial, anti-inflammatory, prebiotic, and anticarcinogenic activity [33].

### Content of Vitamin C

Ascorbic acid is one of the biologically active compounds found in poppy. It is regarded as a powerful antioxidant. The content of vitamin C in fruit and vegetables may differ widely, depending on the plants themselves, the species, cultivar, ripeness, and then also on the losses incurred in storage, transport, processing, and preparation. The degradation of vitamin C is accelerated by high temperature (from as little as 40 °C), presence of oxygen or ions of heavy metals, a neutral or alkaline environment, and sunlight. It is also destroyed by drying, UV radiation, preservatives, and some medications. Significant loss of ascorbic acid is caused by the oxidase family of enzymes, which are released when the cellular structure is ruptured, e.g., during peeling, bruising, or chopping. The activity of those enzymes is significantly reduced in an acidic environment and in low temperatures, e.g., during freezing or fermentation, stopping the degradation of vitamin C. After thermal processing at home, on average, 30–60% of vitamin C is left in fruit and vegetables, but storage of already cooked vegetables, irrespective of the storage temperature (whether at room temperature or in the fridge), incurs even greater losses, even up to approx. 60–80% of vitamin C [34, 35].

Poppy is a very good source of vitamin C. According to Vardavas et al. [31], the content of ascorbic acid in fresh poppy greens amounts to 17 mg/100 g of fresh weight. In another study, the amount found in leaves was similar, reaching 14.11 mg/100 g of fresh weight [32]. The values observed in this study (15–16 mg/100 mL of infusion) indicate that a considerable proportion of vitamin C is transferred into the infusion. This may be due to the use of lyophilization (freeze-drying) to dry the product assuring a minimal loss of the vitamin. From a single glass (250 mL) of the infusion, we will obtain approx. 37–40 mg of vitamin C, which is equivalent to 100 g of lemon, approx. 20 g of parsley leaf, or a little under 30 g of black currant. Considering the above and the daily requirement for vitamin C, which amounts to 75.0 mg per day [36], it may be concluded that the examined poppy flower infusions are a good source of this compound (53% RDA). Please note as well that low-concentration 0.5% infusions were considered in this study.

In addition, no statistically significant differences were noted with regard to the temperature of water used to prepare the infusions. Bearing in mind that L-ascorbic acid is a labile substance, easily degraded in high temperatures, it may be presumed that flowers of the common poppy contain compounds which form complexes with L-ascorbic acid, protecting it against degradation.

### Mineral Content

Macroelements such as potassium, sodium, calcium, magnesium, or phosphorus are essential dietary ingredients which must be supplied to the body to maintain normal bodily functions. The content of potassium and sodium in 0.5% poppy infusions deserves special consideration. Potassium is the main intracellular cation responsible for regulating the normal function of neurons and muscles. Together with sodium ions, it is involved in the function of Na+/K+-ATPase and in creating the electrochemical gradient on both sides of cell membranes. Potassium deficiency may cause muscle weakness and paralysis, whereas an overdose may lead to cardiac arrest and ulcerations of the small intestine [10]. The recommended daily allowance for potassium is approx. 4.7 g [36]. The infusions included in this study were found to contain significantly more K than Na, which may be a beneficial quality for patients with arterial hypertension, who are recommended to reduce their sodium intake in favor of potassium. A glass of a poppy flower infusion, containing approx. 64–72 mg K, covers 1.5% of the daily requirement for that mineral. The same glass of poppy infusion, with approx. 1.3 mg Na, covers approx. 0.2% of the daily requirement for that mineral.

Turning to other macroelements, the infusions contained significantly smaller amounts of Ca, Mg, and P. Still, they can provide an additional dietary source of those minerals. Taking into consideration the average daily requirement for calcium, amounting to approx. 1000–1200 mg, a glass of the infusion provides about 0.4–0.6 mg Ca. It is likewise with magnesium and phosphorus. A glass of a poppy flower infusion supplies 1.6–2.0 mg Mg and 4–5 mg P, while the recommended daily allowance for those minerals amounts to 320–420 mg for magnesium and 700 mg for phosphorus. Brzezicha-Cirocka et al. [37], in their investigation of black tea infusions, obtained higher values for Ca and Mg (respectively, 3.54 mg and 4.39 mg per 200 mL). However, the tea infusions had a higher mass concentration, amounting to 2.0%. Bearing in mind the 0.5% concentration of the poppy infusions, the latter product is richer in Mg than the popular black tea.

The temperature used to prepare the infusions is an important factor influencing the extraction of constituent substances into the solution. For all the macroelements, the lowest concentrations were observed with the temperature of 25 °C, with the exception of Na. The highest concentrations, on the other hand, were observed in infusions made at 70 and 80 °C. In the case of Ca, an upward trend was observed, meaning that its content increased together with the temperature.

It is important to note that for heavy metals and certain trace elements, it is preferred that their content in the diet stays below certain levels, due to the risk of toxic effects if the body is exposed to excessive quantities of those minerals. Microelements such as Mn, Fe, Zn, Cu, Mo, and Si, despite their low levels in the body and the much lower recommended daily allowances compared with those for macroelements, play a vital role in normal body function. The role of some other elements, such as strontium or aluminum, is not known [38].

Poppy plants are capable of accumulating minerals, including heavy metals. According to Kostic et al. [39], poppy flowers are the source of Fe (423.32 mg kg^−1^), Mn (20.61 mg kg^−1^), Zn (31.80 mg kg^−1^), and Cu (35.50 mg kg^−1^).

In the studied infusions, the levels of Cu, Mo, Ni, and Sr, which are required in the body in trace amounts, while their excess is harmful, do not exceed safe limits.

Based on our results, it has been calculated that 1 cup of infusion (200 mL) contains 0.0096–0.013 mg Cu and 0.002–0.0042 mg Mo. According to the Dietary Reference Values for nutrients [36], the demand for Cu is 1.5 mg/day and for Mo, 0.065 mg/day. The amounts of these elements found in 200 mL of the brew cover 0.64–0.87% of the daily Cu requirement and 3–6.5% of the daily Mo requirement.

In terms of nickel, the tolerable upper intake level is 1.0 mg/day of soluble nickel salts [40], and therefore, 1 cup of poppy infusion covers 0.4–0.6% of this amount. A healthy diet should contain 2–4 mg Sr/day [41, 42]. The values obtained in our research indicate that drinking 200 mL of infusion provides 0.0026–0.0032 mg Sr, which covers only a small percentage of the demand for this element (approx. 0.2%).

For the majority of microelements, the highest concentrations were found at the highest temperature (Mn, Cu, Cr, Si, Ni, Al, Fe, Mo), while the lowest at 25 °C (Zn, Mn, Cu, Cr, Si, Ni, Al).

## Conclusions

Poppy petal infusions were characterized by good antioxidant potential, with a considerable content of polyphenols, vitamin C, and many elements. The temperature of the infusion did not affect the content of polyphenols or vitamin C. However, it did significantly affect the antioxidant potential of the infusions. The highest antioxidant activity was observed in the infusion prepared with water at 80 °C. The infusions included in the study contained a number of minerals. No significant effect of temperature was found for the content of K, Zn, Cu, Fe, and Ni in the infusions. On the other hand, the content of Ca in the infusions was significantly correlated with the increasing temperature of the water used to make them. Poppy petal infusions brewed at 80 °C and 90 °C may serve as a valuable dietary supplement, providing antioxidants and minerals required by the human body to function normally.

## Electronic Supplementary Material

ESM 1 (DOCX 14 kb).
