# Supplementary material for: Mineral Composition and Antioxidant Potential in the Common Poppy (Papaver rhoeas L.) Petal Infusions
Source: Biol Trace Elem Res. 2020 Apr 18;199(1):371–81. doi: 10.1007/s12011-020-02134-7 (PMC7746557; doi:10.1007/s12011-020-02134-7)
Supplement: Supplementary file 1 — (DOCX 14 kb). [file 12011_2020_2134_MOESM1_ESM.docx]

Tab. 6 Analysis of reference material Bovine Muscle NIST-SRM 8414 and Bone Meal NIST-SRM 1486* using ICP-OES

| Chemical element | Certified [mg/kg] | Measured [mg/kg] |
| --- | --- | --- |
| Calcium | 145 ± 20 | 130.5709 |
| Chromium | 0.071 ± 0.038 | 0.0875 |
| Aluminum | 1.7 ± 1.4 | 1.2290 |
| Copper | 2.84 ± 0.45 | 2.5820 |
| Iron | 71.2 ± 9.2 | 73.8790 |
| Magnesium | 960 ± 95 | 954.7179 |
| Manganese | 0.37 ± 0.09 | 0.3860 |
| Nickel | 0.05 ± 0.04 | 0.0897 |
| Strontium | 0.052 ± 0.015 | 0.0655 |
| Sodium | 2100 ± 80 | 2149.1556 |
| Phosphorus | 8360 ± 450 | 8864.9444 |
| Potassium | 15170 ± 0.037 | 14831.9658 |
| Zinc | 142 ± 14 | 145.3675 |
| Molybdenum | 0.08 ± 0.06 | 0.1175 |
| Silicon* | <200 | 185.4000 |
